# Berberine Ameliorates Postoperative Cognitive Dysfunction in Aged Mice and Regulates the PI3K‐AKT Pathway: A Network Pharmacology Study

**DOI:** 10.1111/jcmm.70744

**Published:** 2025-07-30

**Authors:** Xuan Li, Shiyu Meng, Jiayi Liu, Meixian Sun, Mao Zhou, Fengtao Ji, Yu Hong

**Affiliations:** ^1^ Department of Anesthesiology Sun Yat‐Sen Memorial Hospital Guangzhou China; ^2^ The Eighth People's Hospital of Qingdao Shandong Province China

**Keywords:** aged mice, berberine, network pharmacology, pI3K‐AKT pathway, postoperative cognitive dysfunction

## Abstract

In this study, we investigated the therapeutic potential of Berberine (BBR), an anti‐inflammatory agent capable of penetrating the blood–brain barrier, for mitigating postoperative cognitive dysfunction (POCD) in aged mice. BBR was administered at a dose of 10 mg/kg daily for 2 weeks and significantly improved cognitive impairments induced by surgical and anaesthesia‐related factors. Specifically, BBR markedly suppressed glial cell activation and reduced levels of pro‐inflammatory cytokines such as tumour necrosis factor alpha (TNF‐α) and interleukin‐1 beta (IL‐1β). Additionally, it alleviated oxidative stress markers and lipid accumulation. Using network pharmacology analysis, we demonstrated that BBR modulates neuroinflammation, oxidative processes and lipid metabolism by inhibiting the phosphorylation of the phosphatidylinositol 3‐kinase (PI3K)/protein kinase B (Akt) pathway. Furthermore, extended research revealed that BBR upregulated the expression of PPAR‐*γ* mRNA, suggesting a neuroprotective mechanism via regulation of the PI3K‐Akt pathway. These findings support the potential application of BBR as a therapeutic agent for managing POCD in elderly populations.

## 1 Introduction

Postoperative cognitive dysfunction (POCD) is commonly identified as a major perioperative complication of the central nervous system in elderly surgical patients, often manifesting as significant changes in personality, reduced social interactions and diminished cognitive abilities [1]. Advanced age is widely acknowledged as a critical independent risk factor that substantially increases the likelihood of POCD development [2]. Epidemiological studies indicate that the prevalence of POCD varies between 10% and 54%, with higher rates predominantly observed in individuals aged 65 years and older [3]. Notably, a subset of these patients may progress to dementia within three to 5 years following the onset of POCD symptoms [4]. The decline in cognitive function associated with surgical interventions significantly impairs quality of life, elevates the risk of additional perioperative complications and contributes to increased mortality rates [5]. Despite extensive research efforts, the exact pathogenic mechanisms underlying POCD remain incompletely understood, with neuroinflammation frequently highlighted as a key contributing factor. Currently, effective therapeutic strategies for addressing this condition are limited, underscoring an important unmet medical need [6]. The preservation of optimal cognitive function largely depends on the stable physiological integrity of neuronal systems.

Berberine (BBR), a naturally occurring isoquinoline alkaloid derived from various medicinal plants, has recently garnered renewed attention in scientific research due to its potential therapeutic benefits for managing neurodegenerative and neuropsychiatric disorders. This resurgence of interest is primarily attributed to BBR's diverse pharmacological effects, including its ability to reduce neuroinflammation, regulate hormonal pathways and modulate neurotransmitter activities, thereby providing promising opportunities for the treatment of complex brain disorders [7, 8, 9, 10]. Extensive studies have confirmed BBR's comprehensive pharmacological profile, which encompasses anti‐inflammatory, antioxidant, antineoplastic and lipid‐modulating properties, contributing to its broad therapeutic potential [11, 12, 13, 14]. Detailed investigations have demonstrated that BBR effectively suppresses the production of pro‐inflammatory mediators by inhibiting toll‐like receptor 4 activation and the subsequent nuclear factor‐κB signalling cascade in experimental models of endotoxaemia [15]. Given its well‐documented ability to cross the blood–brain barrier efficiently [16], there is a strong rationale to explore whether BBR can attenuate inflammatory responses associated with surgical procedures, potentially preventing the onset of POCD. While previous studies have highlighted BBR's neuroprotective effects in conditions such as cerebral ischaemia [17] and Alzheimer's disease [18], its specific role in preventing or treating POCD remains underexplored. The current study aims to investigate whether systemic administration of BBR can effectively alleviate cognitive deficits induced by surgical stress in a model simulating the clinical conditions of POCD in humans. Furthermore, network pharmacology has emerged as a cutting‐edge and integrative discipline that synergistically combines systems biology, bioinformatics and network science to elucidate the complex pharmacological mechanisms of drugs. This approach not only delineates the intricate interactions between drugs and disease pathways but also supports the identification and development of novel therapeutic agents. By examining drug‐disease interactions from a network‐based perspective, network pharmacology offers insights into potential therapeutic targets and critical biological functions, thereby promoting a more profound understanding of drug actions and accelerating the efficiency of drug discovery processes.

This study lays a robust experimental groundwork for future research into the mechanisms by which BBR exerts its preventive and therapeutic effects on POCD. The results suggest that BBR holds considerable promise as a feasible therapeutic candidate for overcoming the challenges associated with POCD, thereby underscoring its potential for development as a pharmacological intervention.

## 2 Materials and Methods

### 2.1 Animals

**FIGURE 1:**
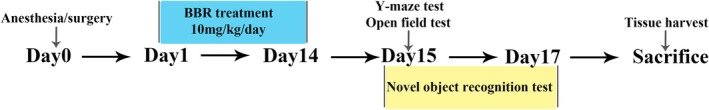
The schematic timeline of the experimental process.

Male C57BL/6 mice, aged 16–18 months and weighing 35–40 g, were provided by Sun Yat‐sen University (Guangzhou, China) for this study. The animals were housed in a specific pathogen‐free (SPF) environment with controlled ambient conditions: a temperature of 20°C–24°C, humidity of 40%–60% and a 12‐h light–dark cycle (light period: 07:00–19:00). Mice were randomly assigned into four groups (*n* = 10 per group): control (CON), control supplemented with BBR (CON + BBR), POCD‐induced group and POCD group treated with BBR (POCD + BBR). Animals were group‐housed (five per cage) with ad libitum access to food and water to ensure their well‐being and minimise stress‐related confounding factors. A two‐week acclimatisation period preceded the experimental procedures to stabilise physiological baselines and reduce environmental stress. Ethical approval for the study was granted by the Institutional Animal Care and Use Committee (Approval No: SYSU‐IACUC‐2023‐000769) and the Laboratory Animal Ethics Committee of Sun Yat‐sen University, ensuring compliance with all applicable guidelines for humane animal care and use. The detailed timeline and sequence of experimental protocols are presented in Figure 1.

### 2.2 Animal Model

The POCD model was successfully established by integrating isoflurane anaesthesia with exploratory laparotomy [19, 20, 21]. Initially, mice were subjected to a 30‐min exposure to an oxygen‐enriched atmosphere containing 2% isoflurane to induce deep anaesthesia. Subsequently, a precise midline incision, approximately 2 cm in length, was performed on the abdomen to facilitate exploration of the abdominal cavity, including organs such as the liver, spleen and intestines. The incision was closed using sterile 6–0 surgical sutures for meticulous closure of both the peritoneum and the outer skin layer. The entire surgical procedure, lasting approximately 30 min, was conducted under continuous isoflurane inhalation anaesthesia. Based on prior studies conducted by our research team, it was confirmed that mice undergoing this surgical intervention maintained normal physiological parameters, including heart rate and respiratory rate, with no evidence of hypoxia [22]. Throughout the procedure, the depth of anaesthesia was rigorously monitored using a GE B450 anaesthesia monitor to ensure complete unresponsiveness to physical stimuli, such as toe pinch. Additionally, body temperature was meticulously controlled at a stable 37°C using a specialised heating blanket (Model 69,020, RWD, CHN) to prevent hypothermia. Mice assigned to the control group did not receive any form of anaesthesia or surgical intervention, thereby preserving their baseline physiological conditions.

### 2.3 Drug

Beginning on the day subsequent to their anaesthesia and surgical procedures, elderly mice assigned to the CON + BBR and POCD + BBR groups were subjected to a regimen involving daily intraperitoneal injections. These injections contained BBR (B414323, Aladdin, CHN) at a standardised dose of 10 mg/kg, administered consistently over a two‐week period. This particular dosage is based on preliminary experiments and previous literature [23]. The BBR used was freshly prepared each day by solubilising the alkaloid powder in sterile normal saline, ensuring maximum effectiveness and stability of the solution. To preserve the integrity and uniformity of the experimental conditions, similarly aged mice in the CON and POCD groups received daily intraperitoneal injections of the same volume of normal saline, serving as the placebo group. This protocol ensured that all variables except the treatment were controlled across the different groups, thereby maintaining the scientific rigour of the study.

### 2.4 Behavioural Studies

Behavioural assessments were conducted in an acoustically isolated chamber from 10:00 to 16:00. Data regarding these behaviours were meticulously documented by two researchers who were not aware of the group assignments of the subjects.

#### 2.4.1 Y‐Maze Test

The Y‐maze apparatus, specifically designed for the assessment of short‐term spatial memory, comprises a central junction with three arms extending at exactly 120° angles relative to one another. In accordance with our experimental protocol, Y‐maze evaluations were conducted precisely 14 days postsurgical intervention. During these assessments, each mouse was allotted a total of 8 min to freely explore the distinct arms of the maze. Throughout this period, detailed records were maintained of all exploratory behaviours exhibited by the mice. A critical behaviour of interest, referred to as ‘alternation behaviour’, was defined as the consecutive entry of each of the three arms without repetition during a single exploratory cycle. To quantify this behaviour, the percentage of alternation was calculated using the formula: Percentage of alternation (%) = (number of alternation behaviours / (total entries–2)) × 100.

#### 2.4.2 Open Field Test

2 weeks after the surgical procedure, each mouse was placed at the centre of a square arena (40 × 40 × 40 cm) with white acrylic walls, as described in previous studies. A 5‐min acclimatisation period was provided to allow each mouse to adapt to the novel environment. Subsequently, locomotor activity, quantified as the total distance travelled, was recorded for 5 min using a high‐resolution video tracking system interfaced with a computer. Offline analysis of locomotor behaviour was performed using the TopScan software (CleverSys, Reston, VA, USA). Between trials, the arena was cleaned with 70% ethanol to eliminate residual odours and contaminants, ensuring a sterile and controlled experimental setup. All assessments were conducted under dim lighting conditions and at consistent times throughout the testing period to minimise stress and maintain standardised testing conditions.

#### 2.4.3 Novel Object Recognition Test

Following the methodologies outlined in prior studies [24, 25], the novel object recognition (NOR) test was administered in a 40 × 40 × 40 cm open field arena, exactly 24 h subsequent to the completion of the open field test (OFT). Initially, to facilitate acclimatisation, each mouse was allowed 5 min in the arena devoid of any objects. During the subsequent training phase, two identical objects were strategically positioned in diagonally opposite corners of the arena, each object being placed 3 cm from the adjacent walls. This setup allowed the mice to freely explore these objects over a 5‐min duration. In the subsequent testing phase, the arena was reconfigured by retaining one of the previously used objects in its original position while substituting the other with a novel object. The exploratory activity directed towards each object was meticulously timed and recorded over a 5‐min interval. Exploration was specifically characterised by the mice either approaching within 1 cm of an object or directly contacting it with their noses. The assessment of exploratory preference was quantified using a discrimination index (DI), defined as DI = T2 / (T1 + T2), where T2 is the time spent exploring the novel object and T1 is the time spent with the familiar object. To ensure the accuracy of subsequent trials, all objects were thoroughly sanitised with 70% ethyl alcohol to remove any lingering scents or contaminants, thus maintaining a controlled environment for accurate behavioural assessment.

### 2.5 Western Blot Analysis

For this study, hippocampal tissues were processed using RIPA lysis buffer (P0013B, Beyotime, China), a common choice for thorough protein extraction. The concentration of the extracted proteins was accurately assessed utilising a BCA protein assay kit (P0010, Beyotime, China), ensuring precise measurement for subsequent analyses. Following protein isolation, samples were subjected to electrophoresis on 10% SDS‐PAGE gels (P0012A, Beyotime, China) to achieve separation based on molecular weight. These proteins were then electroblotted onto PVDF membranes (ISEQ00010, Merck Millipore, USA), which provide a stable and reliable medium for protein transfer. To minimise background noise during detection, the membranes were incubated in a solution of 5% skimmed milk (A600669, Sangon Biotech, China) for 1 h at room temperature, effectively blocking nonspecific protein sites. The primary antibodies, carefully chosen for their specificity, were applied to the membranes and left to bind overnight at 4°C. These antibodies included rabbit polyclonal antimouse TNF‐α (1:1000, AF8208, Beyotime, China), rabbit polyclonal antimouse IL‐1β (1:1000, AF7209, Beyotime, China) and rabbit monoclonal antimouse GAPDH (1:1000, AF1216, Beyotime, China), each targeted to identify specific cellular markers. Postprimary incubation, the membranes were treated with horseradish peroxidase (HRP)‐conjugated goat antirabbit IgG (1:5000, A0208, Beyotime, China) at room temperature for 1 h, facilitating the enzymatic detection of the primary antibody complexes. Visualisation of protein bands was conducted using an enhanced chemiluminescence (ECL) reagent (WBKLS0100, Merck Millipore, USA), providing high‐sensitivity detection of protein expressions. The bands were then quantitatively analysed using ImageJ software (National Institutes of Health, Bethesda, MD, USA), allowing for detailed analysis and comparison of protein levels across samples. This protocol ensured a rigorous approach to the qualitative and quantitative analysis of specific protein markers within hippocampal tissue, crucial for investigating cellular mechanisms linked to neurological health and disease.

### 2.6 Immunofluorescence Assay

Brain tissues were preserved using OCT compound and sectioned into slices with a thickness of 25 μm. These sections were subjected to triple washing with phosphate‐buffered saline (PBS) to remove any residual OCT compound from their surfaces. Subsequently, the sections were incubated with goat serum (Catalogue No. 16210072, Gibco, United States) for 1 h at room temperature to block nonspecific binding sites. The tissue samples were then incubated overnight at 4°C with a rabbit polyclonal anti‐GFAP antibody raised in mouse (dilution 1:100, Catalogue No. BA0094, Boster, China) for specific staining. Following a 1‐h rewarming period at room temperature, the sections underwent another triple wash with PBS and were treated with donkey antimouse IgG (H + L) highly cross‐adsorbed secondary antibody conjugated with Alexa Fluor 647 (dilution 1:1000, Catalogue No. A‐31571, Invitrogen, United States) and CY3‐labelled goat antimouse IgG (dilution 1:500, Catalogue No. A0562, Beyotime, China) for 2 h at room temperature. Nuclei were stained with DAPI (Catalogue No. G1012, Servicebio, China) for 20 min at room temperature. After staining, the sections were sealed with an antifluorescence quenching agent (Catalogue No. P0128M, Beyotime, China) to preserve fluorescence. High‐resolution images of these sections were acquired using a laser confocal microscope at 200 × magnification (Zeiss LSM 800 with Airyscan, Germany).

### 2.7 Oil Red O Staining

Wash the prepared brain sections three times with PBS for 5–10 min each time, followed by washing in 60% isopropanol for 5 min. After washing, transfer the sections to oil red O staining solution for 15 min, avoiding light. Then soak in 60% isopropanol for 1 min, wash with distilled water and place the sections in haematoxylin for 5 min to stain the cell nucleus. After washing with triple distilled water again, rapidly differentiate with 1% hydrochloric acid alcohol for 1–2 s and then slowly wash with flowing water for 1–3 h to restore the cell nucleus to blue. Finally, seal the stained sections with glycerol gelatin and place them under the microscope for observation.

### 2.8 Real‐Time Quantitative Polymerase Chain Reaction(RT‐qPCR)

Total RNA was extracted from frozen hippocampal tissue using the Trizol method, followed by reaction in a pre‐configured reverse transcription system to obtain cDNA. Prepare the system using the SuperReal PreMix kit instruction (FP206‐01, TIANGEN, China) and perform qPCR reaction detection using the Roche LightCycler 480 II Real Time PCR System (Catalogue No. 05015278001, Roche Diagnostics, Germany). The mixed reaction system was preheated at 95°C for 15 min followed by 40 cycle stages: 60°C for 20 s after 95°C for 10 s. Then followed by 1 cycle stage: 72°C for 30 s,95°C for 15 s and finally,60°C for 30 s. The mRNA expressions of PPAR‐γ in hippocampal tissues were detected by 2^−ΔΔCT^ assays with GAPDH as internal references. The required primers were synthesised by Beijing Genomics institution (Forward primer sequence 5′‐3’:GGAGCCTAAGTTTGAGTTTGCTGTG, Reverse primer sequence 5′‐3’:TGCAGCAGGTTGTCTTGGATG).

### 2.9 ROS Evaluation

The concentrations of reactive oxygen species (ROS), malondialdehyde (MDA) and orgotein superoxide dismutase (SOD) were quantified using a specialised Lipid Peroxidation Assay Kit (G1706, G4300, GM1133; Servicebio, China). The assay was performed in strict accordance with the manufacturer's protocol. The activities of ROS, MDA and SOD were precisely determined by measuring the absorbance at a wavelength of 532 nm using a microplate reader (TECAN Spark 10 M, China). The results were normalised to protein content and expressed as units per milligram of protein to ensure data consistency and comparability.

### 2.10 Methodology for Network Pharmacological Analysis of BBR's Mechanism in POCD

Initially, the three‐dimensional structural data of BBR (BBR) were retrieved in SDF format from the PubChem database (https://pubchem.ncbi.nlm.nih.gov/) [26]. This structural file was subsequently submitted to the PharmMapper platform (http://www.lilab‐ecust.cn/pharmmapper/) [27] for systematic target prediction. To ensure comprehensive target identification, complementary searches were conducted using two additional resources: the Traditional Chinese Medicine Systems Pharmacology Database (TCMSP, https://www.tcmsp‐e.com/) [28] and the SwissTargetPrediction database (http://www.swisstargetprediction.ch/) [29].

Following target acquisition, all identified proteins underwent deduplication and standardisation through the UniProt database (https://www.uniprot.org/) [30], with strict species filtering limited to * *Homo sapiens* *. Concurrently, disease‐associated targets for POCD were systematically curated from GeneCards (https://www.genecards.org/) [31]. Using the search terms ‘Postoperative Cognitive Dysfunction’ and its acronym ‘POCD’.

The intersection between BBR's putative targets and POCD‐related targets was determined through Venn analysis, yielding candidate targets for further investigation. These overlapping targets were then subjected to protein–protein interaction (PPI) network construction via the STRING database (https://string‐db.org) [32] under stringent parameters: human species restriction, interaction confidence score ≥ 0.400 and exclusion of isolated nodes.

The resultant network was visualised and topologically analysed using Cytoscape 3.8.0 (https://cytoscape.org) [33], from which the 30 most topologically significant hub targets were identified based on betweenness centrality and degree values. Functional annotation of these core targets was performed through Gene Ontology (GO, http://geneontology.org) [34, 35, 36] enrichment analysis across three domains: biological processes, molecular functions and cellular components. Parallel Kyoto Encyclopedia of Genes and Genomes (KEGG https://www.kegg.jp) [37] pathway analysis was conducted to elucidate relevant signalling pathways. All enrichment results were graphically represented using customised R scripts (ggplot2 package) to generate detailed bubble plots.

### 2.11 Statistical Analysis

The summarised data for all experimental outcomes were systematically presented as mean ± standard deviation (S.D.) Comprehensive statistical analyses were conducted using GraphPad Prism version 9.5 (San Diego, CA, USA), a robust software tool renowned for its analytical capabilities, supplemented by R software to ensure rigorous data integrity and reliability. To assess inter‐group differences, a one‐way repeated measures analysis of variance (ANOVA) was employed, followed by Tukey's post hoc test to evaluate significant pairwise differences among groups. Specifically, the dataset obtained from Y‐maze training sessions underwent a detailed statistical evaluation using two‐way repeated measures ANOVA, which facilitated an in‐depth exploration of both main effects and interactions. Subsequently, Tukey's test was applied to identify and confirm statistically significant discrepancies across experimental groups. Throughout these analyses, a *p*‐value threshold of less than 0.05 was established as the criterion for determining statistical significance, providing a reliable basis for interpreting meaningful variations within the collected data.

## 3 Results

### 3.1 BBR Ameliorated Postoperative Cognitive Dysfunction Caused by Anaesthesia and Surgery in Aged Mice

**FIGURE 2:**
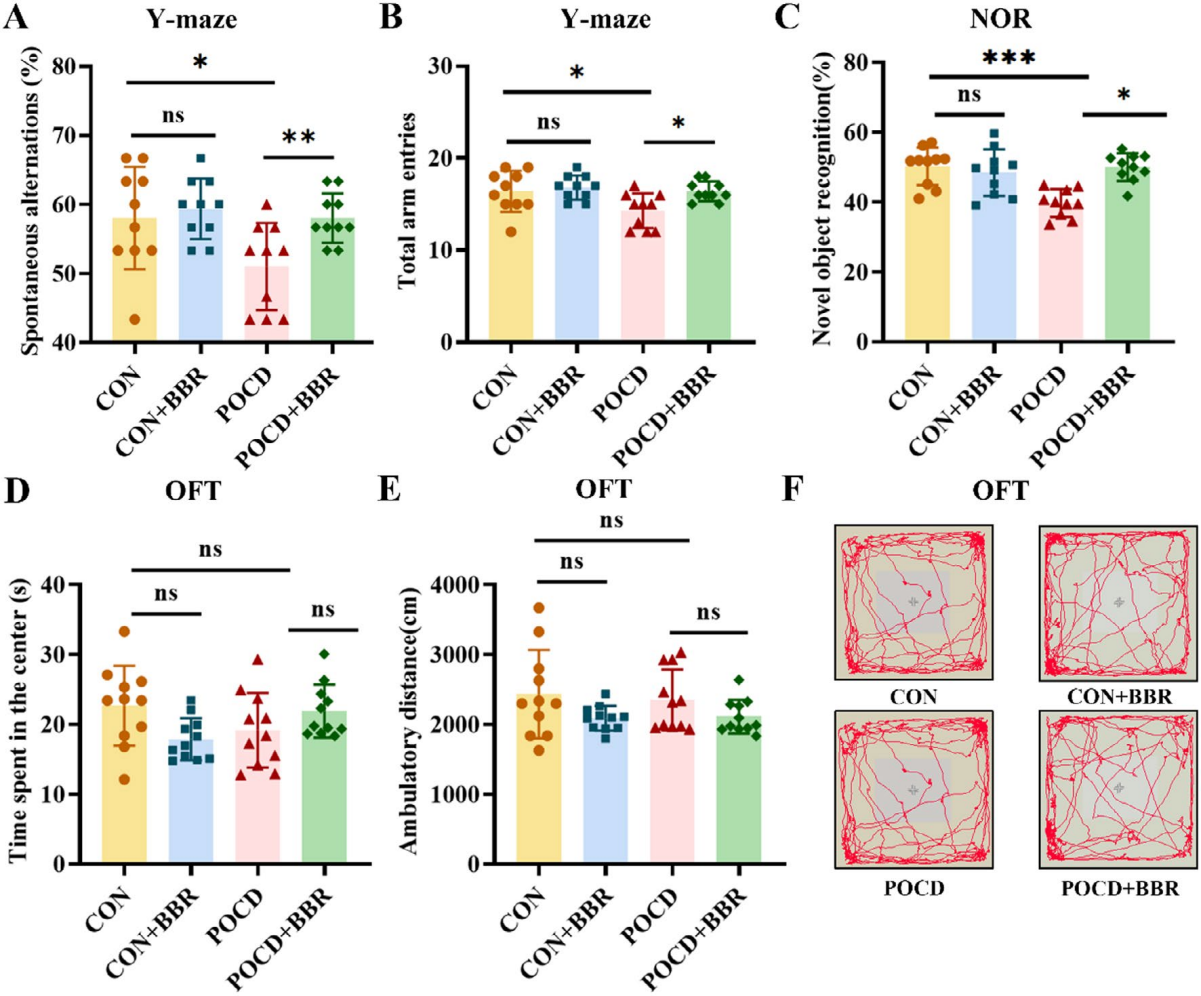
BBR improves spatial cognition and memory abilities in POCD mice. (A, B) Y‐maze test shows that BBR treatment significantly improved spontaneous alternations and total arm entries in POCD mice. (C) The new object recognition experiment shows that BBR improves memory function in POCD mice. (D, E) The open field experiment shows that anaesthesia/surgery did not affect the activity and exploration ability of mice. (F) The movement trajectory of mice in the open field experiment. The findings were presented as mean ± SD, *n* = 10, *p < 0.05, **p < 0.01, ***p < 0.001.

In this study, the Y‐maze, Open Field Test (OFT) and Novel Object Recognition (NOR) test were utilised to assess spatial memory and anxiety‐related behaviours in mice. Mice with POCD demonstrated a significant decrease in spontaneous alternation behaviour within the Y‐maze following anaesthesia and surgery, indicating a marked impairment in spatial learning ability (*p* < 0.05, Figure 2A). However, treatment with BBR was found to effectively reverse these learning deficits, significantly enhancing cognitive performance in these mice. Furthermore, during the Open Field Test, no statistically significant differences were observed in parameters such as total distance travelled or time spent in the centre area across all experimental groups. This suggests that neither anaesthesia nor surgical procedures adversely affected motor coordination or emotional states in the mice (Figure 2D–F). The Novel Object Recognition test, which capitalises on the natural tendency of mice to preferentially explore novel objects over familiar ones, revealed that cognitive functions were negatively impacted by isoflurane exposure and surgical interventions. Nevertheless, BBR treatment significantly attenuated these adverse effects. Specifically, the recognition index of the POCD group was considerably lower than that of the control group (*p* < 0.01), reflecting a reduced capacity to discriminate between familiar and novel objects. In contrast, the administration of BBR markedly increased the recognition index in the POCD+BBR group, surpassing even the levels observed in the control group (*p* < 0.01, Figure 2B,C). Collectively, these findings highlight the substantial cognitive and memory impairments associated with POCD in mice and underscore the therapeutic potential of BBR in mitigating these deficits.

### 3.2 Network Pharmacology Uncovered the Targets and Signalling Pathways Regulated by BBR

**FIGURE 3:**
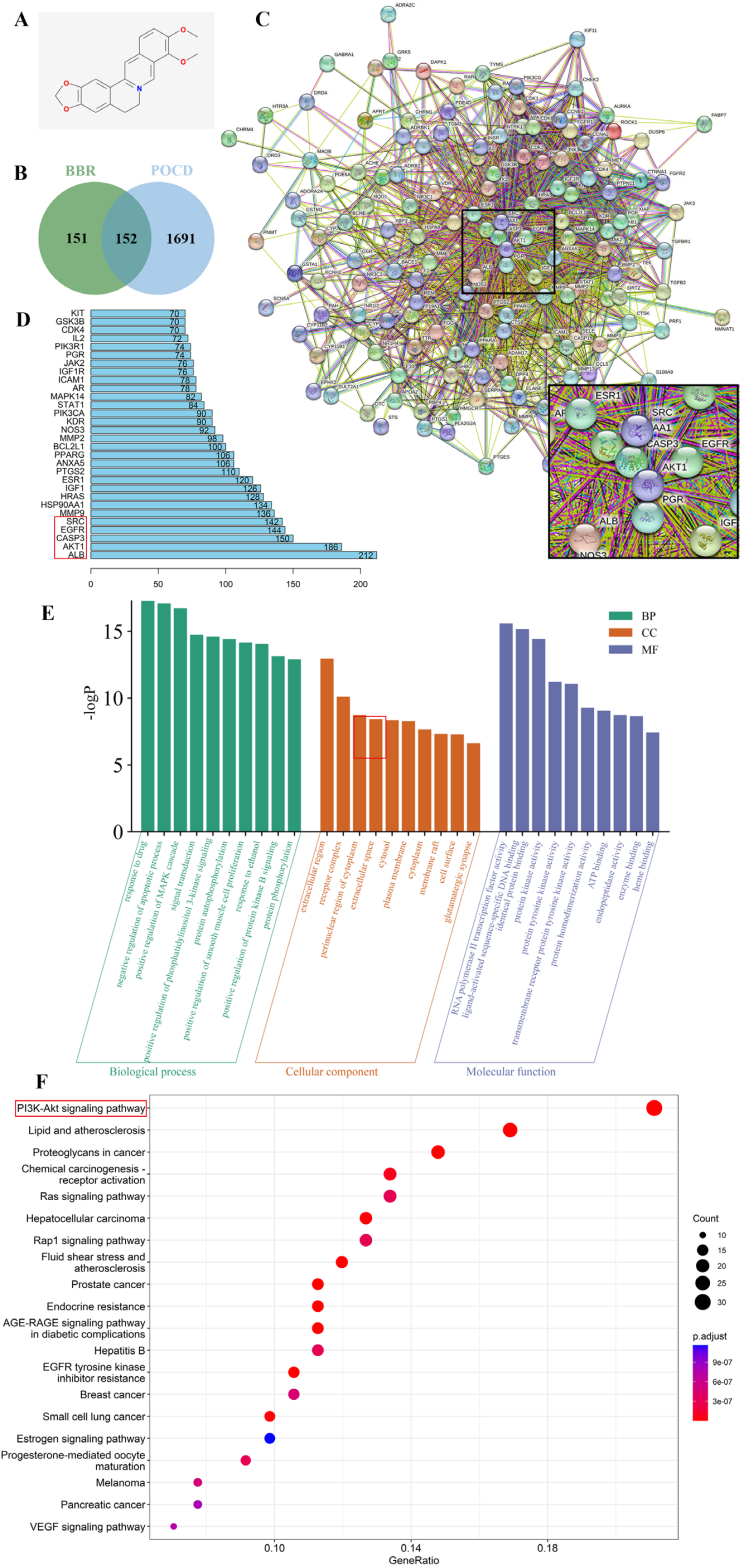
Analysis of network pharmacology for BBR in POCD management. (A) The molecular structure of BBR. (B) Intersection of targets between BBR and POCD‐related disease markers, where green, purple and grey circles denote BBR‐specific, POCD‐specific and common targets respectively. (C) Protein–protein interaction (PPI) network highlighting BBR's involvement in POCD treatment. (D) Illustrates the top 30 targets within the PPI network. (E) Gene Ontology (GO) enrichment analysis for common targets of BBR in POCD, detailing the top 10 enriched results across biological processes (BP), molecular functions (MF) and cellular components (CC). (F) Pathway analysis via KEGG, where circle size correlates with gene count and colour intensity reflects lower P‐values, underscoring significant pathway involvement.

The improvement of BBR on POCD‐related behavioural symptoms has attracted significant attention. To comprehensively investigate the mechanism by which BBR acts on POCD, we employed a network pharmacology approach to elucidate the underlying molecular mechanisms. Network pharmacology serves as a systematic research methodology for exploring drug‐disease relationships and plays a crucial role in identifying potential targets and pathways. Initially, we systematically aggregated 303 potential targets of BBR from multiple authoritative databases, including PharmaMapper, Swiss Target Prediction and TCMSP. Simultaneously, our comprehensive search in the GeneCards database identified 1691 disease‐related targets associated with POCD. Through a rigorous comparative analysis of these datasets, we pinpointed 152 overlapping targets, which were implicated as key mediators in BBR's potential therapeutic effects on POCD (Figure 3B). Subsequently, we constructed a protein–protein interaction (PPI) network for these 152 targets using data from the STRING database, as depicted in Figure 3C. This network was further analysed using Cytoscape, where clustering algorithms highlighted the top 30 significant targets, presented in Figure 3D. Gene Ontology (GO) enrichment analysis elucidated the functional roles of these targets, demonstrating that BBR's mechanisms of action on POCD encompass various levels, including biological processes (BP), cellular components (CC) and molecular functions (MF). Notably, these activities involved drug response, localisation in the extracellular region and RNA polymerase II transcription factor activity, particularly emphasising light‐activated sequence‐specific DNA binding (Figure 3E). Additionally, KEGG pathway analysis was performed to identify the primary signalling pathways modulated by BBR, revealing the top 20 enriched pathways. This analysis confirmed that the PI3K‐Akt signalling pathway, lipid metabolism and atherosclerosis and proteoglycans in cancer are among the most significantly enriched pathways involved in the regulation of POCD by BBR (Figure 3F). This comprehensive network pharmacology analysis not only highlights the PI3K/Akt pathway as a critical mediator through which BBR exerts its effects on POCD but also lays the groundwork for empirical validation. Consequently, based on the findings from the network pharmacology analysis, we propose that BBR may alleviate neuroinflammation and lipid deposition in POCD. To verify this hypothesis, we performed mechanism‐related experimental investigations in these areas.

### 3.3 BBR Suppressed Glia Activation in Aged Mice With Postoperative Cognitive Dysfunction

**FIGURE 4:**
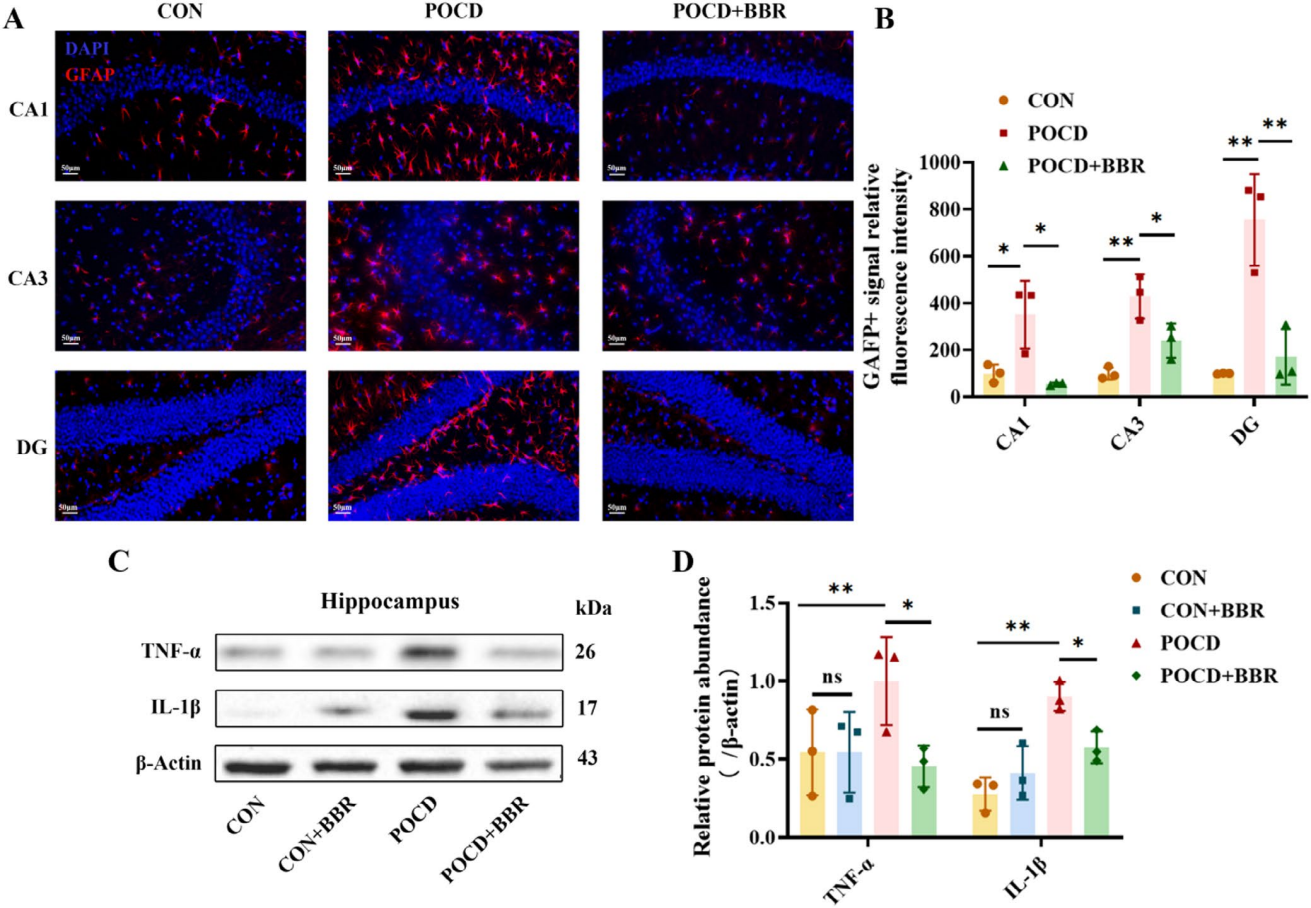
BBR efficacy in reducing glial activation and the secretion of inflammatory cytokines in the hippocampus following anaesthesia and surgical interventions. (A) Utilises immunofluorescence staining to measure expression levels of GFAP across hippocampal regions CA1, CA3 and dentate gyrus (DG). Magnification: 400×. Scale bar = 50 μm. (B) Quantification of the GFAP‐positive fluorescence intensity in CA1, CA3 and DG areas. (C, D) Display the results of western blot analyses detailing the relative concentrations of TNF‐α and IL‐1β proteins in the hippocampus. Statistical values are expressed as mean ± SD, *n* = 3, significance indicated by *p < 0.05, **p < 0.01.

In this study, the activity of astrocytes within the hippocampus was quantitatively assessed by measuring the fluorescence intensity of GFAP immunofluorescence staining. A significant increase in the number of GFAP‐positive astrocytes was observed in the CA1, CA3 and dentate gyrus (DG) regions of the hippocampus following anaesthesia and surgical intervention. Notably, the administration of BBR effectively reversed these changes, significantly attenuating the elevated levels of activated astrocytes (Figure 4A,B). Further investigation into the inflammatory cytokine profiles within the hippocampus revealed a marked upregulation of TNF‐α and IL‐1β protein levels in the POCD group, indicating a pronounced inflammatory response induced by the surgical procedures and anaesthesia. In stark contrast, treatment with BBR substantially mitigated these inflammatory markers, restoring them to levels comparable to those observed in the control group (Figure 4C,D). These findings collectively demonstrate the robust anti‐inflammatory effects of BBR, suggesting its potential to suppress neuroinflammation by modulating astrocyte activity in the hippocampus of aged mice. These results further highlight the therapeutic promise of BBR in alleviating the adverse cognitive effects associated with surgical and anaesthetic interventions through the reduction of hippocampal inflammation.

### 3.4 BBR Inhibited Lipidosis and Oxidative Accumulation in POCD Mice

**FIGURE 5:**
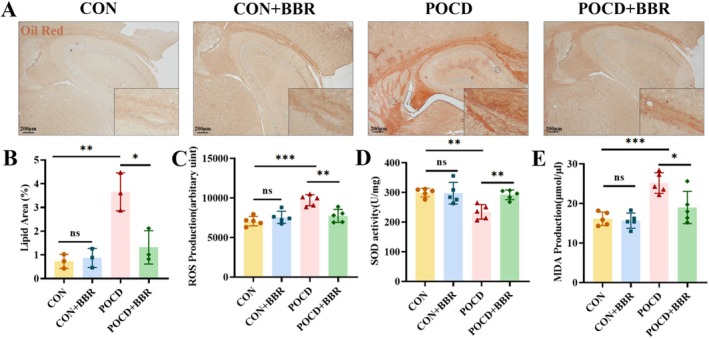
Reduction of lipid accumulation and mitigation of oxidative stress in POCD mice by BBR. (A) Displays Oil Red O staining within the hippocampal region. Magnification: 40×. Scale bar = 200 μm. (B) The statistical chart of lipid accumulation region in oil red staining showed that lipid deposition increased significantly in POCD group and BBR inhibited lipid deposition, *n* = 3. (C‐E) Quantification of hippocampal ROS, SOD and MDA levels, *n* = 5. Statistical values are given as mean ± SD, significance denoted by *p < 0.05, **p < 0.01, ***p < 0.001.

Emerging evidence indicates that the pathogenesis of POCD is intricately linked to both inflammatory responses [38] and oxidative stress mechanisms [39]. Given the recognised antioxidant properties of BBR, we investigated its effects on oxidative stress markers, including reactive oxygen species (ROS), superoxide dismutase (SOD), and malondialdehyde (MDA), within the hippocampus. Our findings demonstrated a significant elevation in these oxidative markers following anaesthesia and surgical procedures. Notably, BBR administration significantly attenuated the increased levels of oxidative stress in the hippocampus, thereby validating our hypothesis regarding BBR's neuroprotective potential against oxidative damage (Figure 5C–E).

Additionally, current studies suggest that excessive lipid accumulation may induce mitochondrial stress, which could subsequently lead to neuronal injury and apoptosis. In this regard, we assessed lipid accumulation in the hippocampus using Oil Red O staining techniques. The results revealed that anaesthesia and surgical procedures significantly enhanced lipid deposition within the hippocampus. Conversely, BBR treatment markedly attenuated this lipid accumulation (Figure 5A,B). The precise mechanisms underlying the contribution of anaesthesia and surgery to increased lipid deposition in the hippocampus remain unclear and warrant further investigation.

### 3.5 BBR inhibited the overactivation of PI3K/AKT pathway in aged mice with POCD

**FIGURE 6:**
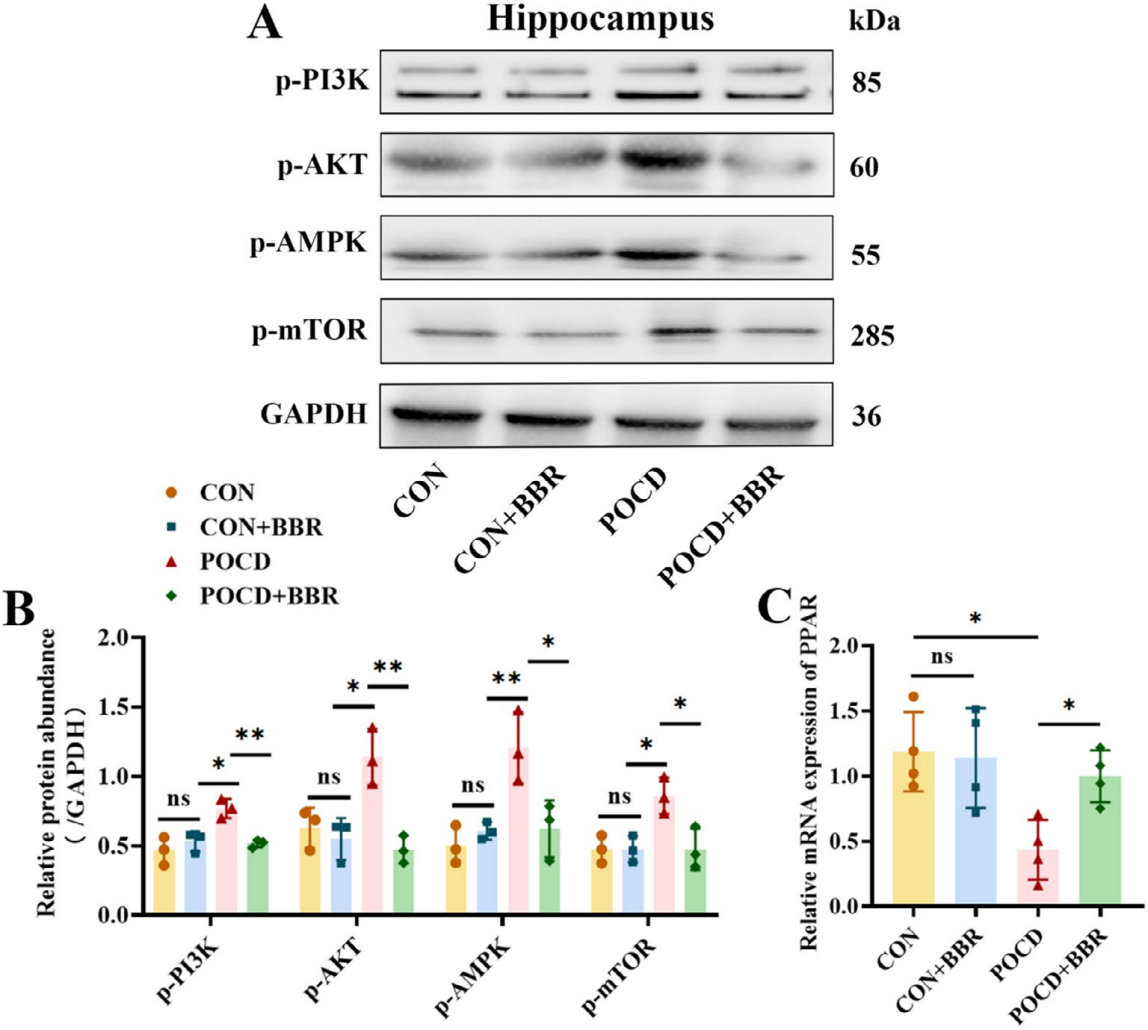
Modulation of the PI3K/AKT/AMPK/mTOR pathway by BBR in POCD. (A) Depicts a typical image showcasing protein levels related to the PI3K/AKT/AMPK/mTOR pathway in each group; (B) Illustrates the protein expression ratios of p‐PI3K/GAPDH, p‐AKT/GAPDH, p‐AMPK/GAPDH and p‐mTOR/GAPDH in each group, *n* = 3. (C) Representative graph showing the relative mRNA levels of the PPAR‐γ, *n* = 4. Data are expressed as mean ± SD, significance levels are indicated as * for *p* < 0.05, ** for *p* < 0.01 and *** for *p* < 0.001.

**FIGURE 7:**
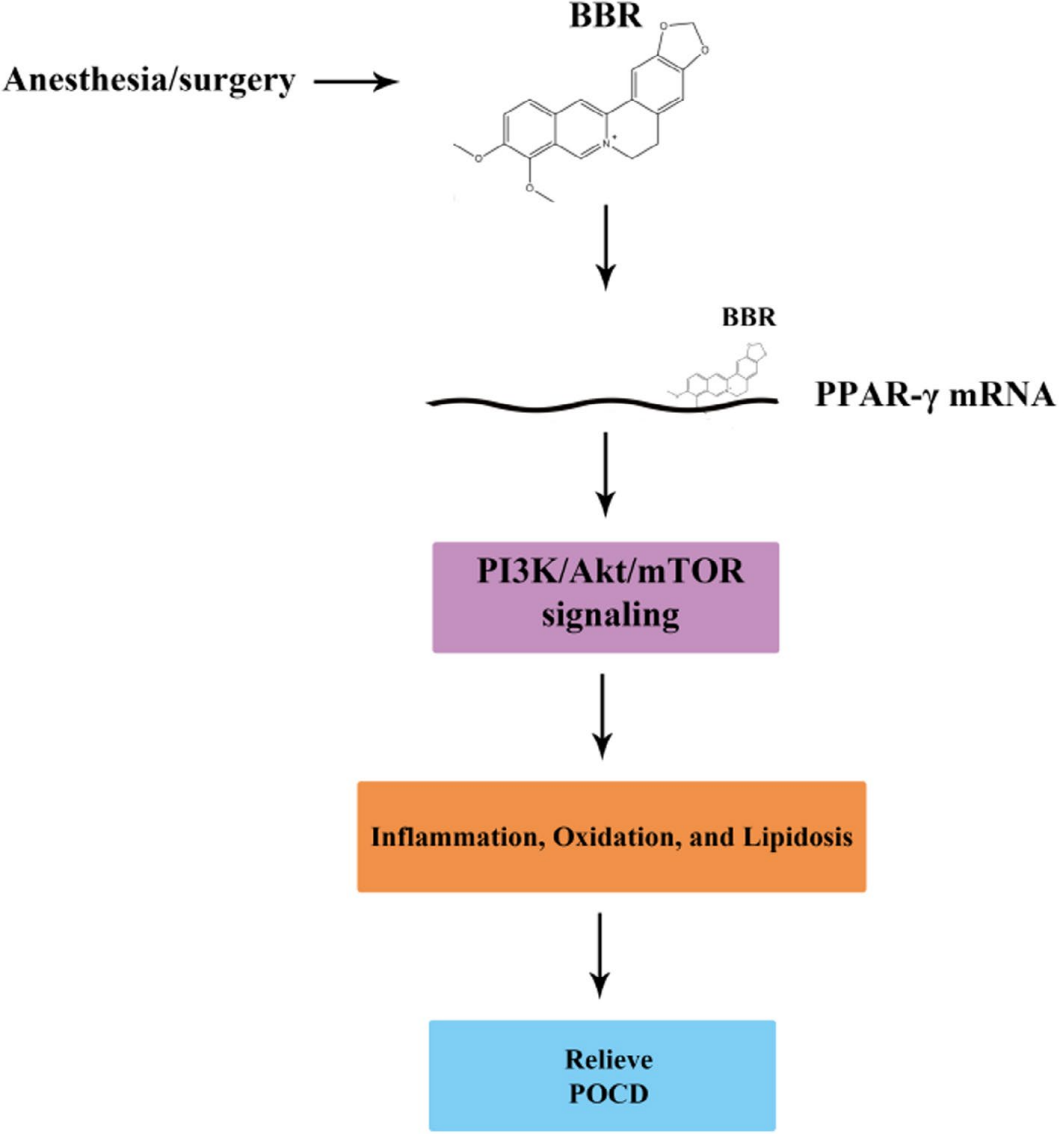
The molecular mechanism by which BBR alleviates the progression of POCD involves multiple pathways. The BBR plays a protective role in preventing hippocampal injury in mice induced by surgery and anaesthesia. Specifically, BBR activates PPAR‐γ mRNA expression, which subsequently mitigates neuroinflammation, oxidative stress and lipid metabolism disorders via the activation of the PI3K/Akt/mTOR signalling pathway.

Network pharmacology analysis revealed that BBR could potentially alleviate the symptoms of POCD by modulating the PI3K/AKT signalling pathway. The PI3K/AKT/mTOR pathway is a critical regulator of diverse cellular functions and has been increasingly acknowledged for its central role in inflammatory diseases [40, 41, 42]. In our recent investigation, Western blot analysis demonstrated that anaesthesia and surgery markedly activated the PI3K/AKT/mTOR pathway in affected cells. Notably, this activation was effectively reversed upon BBR administration, as illustrated in Figures 6 and 7. These results further substantiate the hypothesis that BBR may mitigate POCD symptoms through regulation of this specific signalling pathway. Peroxisome proliferator‐activated receptors (PPARs), a group of nuclear receptor proteins, play a pivotal role in metabolic regulation, inflammation control and oxidative stress management [43, 44]. As an upstream regulator of the PI3K/AKT/mTOR pathway, PPARs exert significant influence over these physiological processes. Our findings indicated that at the mRNA level, PPAR expression was substantially downregulated in the POCD group, while BBR treatment significantly restored this reduction.

## 4 Discussion

The POCD is a clinically significant neurocognitive disorder that frequently affects elderly patients following surgical procedures. It manifests through a spectrum of symptoms, including psychosis, anxiety, changes in personality and impaired memory function. Notably, POCD exhibits a high prevalence after cardiac surgeries as well as various noncardiac interventions, such as abdominal and thoracic surgeries [45]. Furthermore, age has been consistently identified as a critical independent factor influencing both the onset and progression of POCD [46]. Studies indicate that individuals aged 60 years or older experience POCD at rates ranging from 10% to 62%, significantly increasing their risk of developing Alzheimer's disease and progressing to dementia [47]. In this study, we modelled POCD by performing an exploratory laparotomy combined with isoflurane inhalation anaesthesia on 18‐month‐old male C57BL/6 mice. Subsequent behavioural assessments revealed that these anaesthetic and surgical procedures substantially disrupted spatial learning and memory, affecting key hippocampus‐dependent memory functions in both short‐term and long‐term capacities. Despite these pronounced cognitive impairments, our findings also demonstrated that these procedures did not significantly affect locomotor activity or induce notable postoperative anxiety‐related behaviours in the subjects.

Berberine, a potent isoquinoline alkaloid extracted from various traditional Chinese medicinal plants, is widely recognised for its diverse therapeutic properties, particularly in the management of metabolic disorders, the inhibition of microbial infections and the treatment of inflammatory conditions [48, 49, 50]. This compound has been extensively studied, revealing a wide range of pharmacological and biological activities, including, but not limited to, anti‐inflammatory, antioxidant, antitumour and cholesterol‐lowering effects [51, 52, 53, 54]. In this study, we specifically investigated BBR's therapeutic effects on hippocampal‐dependent cognitive functions, which are frequently impaired by surgical and anaesthetic interventions in elderly patients. Our results indicate that BBR significantly mitigates memory impairments associated with these medical procedures, thereby enhancing hippocampus‐related cognitive functions. Furthermore, our findings corroborate previous studies demonstrating that in aged mice not exposed to surgical stress, BBR does not induce behavioural changes, reinforcing its safety and efficacy. Collectively, these findings support the role of BBR in alleviating POCD in elderly mice, underscoring its potential as a valuable therapeutic agent for promoting cognitive recovery following surgery.

Network pharmacology serves as an integrative methodology aimed at elucidating complex signalling perturbations and the modalities of drug actions within the context of multifaceted diseases. This approach has become a cornerstone in uncovering the intricate and precise mechanisms underlying drug functionality, thereby enhancing our comprehension of therapeutic interactions and effects [55, 56]. In this study, we employed network pharmacology to systematically explore the synergistic effects and detailed mechanisms of BBR. Through the application of this sophisticated analytical framework, we successfully identified the active constituents of BBR and clarified their interactions within biological pathways, providing a holistic understanding of BBR's molecular‐level operations. This investigation not only confirmed the robust protective effects of BBR against POCD but also delineated the specific molecular pathways through which BBR exerts its beneficial influences, thus enriching our insight into its potential therapeutic applications.

Network pharmacology has pinpointed the PI3K/Akt signalling pathway as a potentially critical mediator through which BBR may exert its therapeutic effects on POCD. To validate this potential mechanism, our research team embarked on a series of experiments aimed at examining this pathway more closely. Our studies have confirmed that BBR modulates the PI3K/Akt signalling pathway, potentially accounting for its effects in mitigating oxidative stress, neuroinflammation and lipid accumulation, which are common contributors to POCD. Further insights into BBR's mechanism suggest that its protective impact on POCD may be linked predominantly to the inhibition of the PI3K/AKT/mTOR pathway, a pathway that has been implicated in a variety of disease states including acute lung injury, osteoarthritis and ischaemic diseases [40, 57, 58, 59]. Extensive research has demonstrated that the PI3K enzyme plays a vital role in regulating macrophage polarisation and cell proliferation, crucial processes in the body's response to inflammation [60, 61]. As a key intracellular signalling molecule, PI3K binds to Akt and subsequent phosphorylation levels of PI3K and Akt have been closely associated with inflammatory disease progression [62]. The activation of Akt stimulates downstream signalling including the activation of mTOR, a critical regulator of cell growth and metabolism [63, 64, 65]. Given these insights, targeting the PI3K/AKT/mTOR signalling pathway has been pursued as a therapeutic strategy in recent clinical studies focusing on POCD [66, 67, 68]. Our experimental findings have shown an increase in the phosphorylation levels of PI3K, AKT and mTOR in the hippocampi of models with POCD, indicating an upregulation of this pathway. Treatment with BBR effectively reduced the phosphorylation levels of p‐PI3K, p‐AKT and p‐mTOR, demonstrating that BBR's therapeutic action in mitigating POCD likely involves the suppression of the PI3K/AKT/mTOR pathway. This confirms that BBR not only modulates this key signalling pathway but also provides a significant neuroprotective effect in the context of postoperative cognitive impairments.

Various mechanisms are implicated in the pathogenesis of POCD, including neuroinflammation, oxidative stress and neurodegeneration [69, 70, 71]. Glial cells, particularly microglia and astrocytes, have been shown to mediate neuroinflammation via the NF‐κB and Toll‐like receptor pathways, which are critical components of the inflammatory response [72, 73, 74]. Consequently, inhibiting glial cell activation to reduce neuroinflammation and oxidative stress may represent a promising therapeutic strategy for POCD. In this study, our network pharmacology analysis indicated that BBR could suppress neuroinflammation, oxidative stress and lipid metabolism disorders by modulating the PI3K/AKT signalling pathway. Consistent with these findings, our biological experiments confirmed that BBR significantly downregulated pro‐inflammatory cytokines and oxidative stress markers while effectively suppressing glial cell activation in vivo. Targeting these cellular pathways to inhibit glial cell activation represents a promising therapeutic strategy for alleviating neuroinflammation and oxidative stress, which may potentially mitigate the cognitive impairments associated with POCD. In our comprehensive study, network pharmacology analyses revealed that BBR could modulate the PI3K/AKT signalling pathway, thereby suppressing neuroinflammation, reducing oxidative damage and inhibiting lipidosis. Furthermore, our biological assays corroborated these network pharmacology insights by demonstrating that BBR significantly diminishes pro‐inflammatory cytokines and oxidative stress markers. Additionally, it markedly suppresses the activation of glial cells in vivo. These experimental findings validate the mechanisms hypothesised through our pharmacological network analysis and highlight the therapeutic potential of BBR in addressing the underlying pathophysiological processes of POCD, thus positioning it as a promising candidate for further clinical investigation.

In our study, we identified BBR as a potential PPAR‐γ agonist that may contribute to the alleviation of POCD. Peroxisome proliferator‐activated receptors (PPARs) comprise a family of nuclear receptor proteins that play essential roles in regulating metabolism, inflammation and oxidative stress [43, 44], which is a molecule located upstream of AKT [75]. Notably, PPAR‐γ, a key member of the PPAR family, is instrumental in reducing inflammatory responses [76]. Extensive research has demonstrated that the activation of PPAR‐γ significantly enhances neuroprotection against neuroinflammation, highlighting its promising therapeutic implications [77, 78, 79, 80].

However, the mechanism by which BBR acts on POCD is both complex and multifaceted. In addition to the PI3K/Akt signalling pathway, network pharmacology analysis indicates that other pathways, such as the AGE‐RAGE signalling pathway involved in diabetic complications, may also play a potential role. The AGE‐RAGE signalling pathway reportedly activates tyrosine‐protein kinase JAK2 (JAK2), mitogen‐activated protein kinase (MAPK), nuclear factor NF‐kappa‐B (NFκB) and protein kinase C (PKC), thereby inducing inflammation and oxidative stress [81]. Furthermore, the AGE‐RAGE signalling pathway has been found to directly compromise blood–brain barrier permeability [82]. These findings suggest that BBR may alleviate inflammation and oxidative stress via the AGE‐RAGE signalling pathway, thereby improving cognitive function—a direction that warrants further exploration in future studies. Additionally, although we observed that BBR enhances the behavioural performance of POCD mice and inhibits the activation of the PI3K/AKT/mTOR pathway, confirmatory experiments are still necessary. For instance, it would be important to investigate whether the therapeutic effects of BBR are altered upon blocking the PI3K/AKT/mTOR pathway, thus further establishing the causality of these phenomena and validating our hypothesis. In addition, it should be noted that in our current experiment, only male mice were used, which is also one of our limitations. In fact, relevant studies have found that oestrogen can affect cognitive changes in mice after surgery [83]. Moreover, some pharmacological effects of BBR seem to be influenced by oestrogen. According to previous studies, BBR may have a better effect on improving glucose metabolism in women [84]. Therefore, in order to avoid any interference from oestrogen, based on multiple studies [85, 86, 87], we chose to use male mice in this study, but this may lead us to ignore the influence of gender differences. In future research, considering female mice as controls to observe the relationship between BBR and POCD may lead to unexpected and interesting findings.

## 5 Conclusion

The study demonstrated that BBR alleviates cognitive decline and memory impairments by attenuating neuroinflammation‐related and oxidative stress‐associated responses in the hippocampus. Specifically, through the upregulation of PPAR‐γ mRNA expression and the downregulation of the PI3K/AKT signalling pathway, BBR effectively mitigates oxidative damage, neuroinflammation and lipid accumulation induced by surgical interventions and anaesthesia (as shown in Figure 7). These findings suggest that BBR holds significant potential as a therapeutic candidate for the prevention of POCD.

## Availability of Data and Materials

All the data supporting our findings are contained within the manuscript.

## Author Contributions

**Xuan Li:** conceptualization (equal), investigation (equal), methodology (equal). **Shiyu Meng:** validation (equal), writing – original draft (equal). **Jiayi Liu:** validation (equal), writing – original draft (equal). **Meixian Sun:** data curation (equal). **Mao Zhou:** supervision (equal). **Fengtao Ji:** supervision (equal). **Yu Hong:** conceptualization (equal), investigation (equal), methodology (equal).

## Ethics Statement

All the animal experiments in the present study were approved by the Institutional Animal Care and Use Committee (Approval No: SYSU‐IACUC‐2023‐000796) and the Laboratory Animal Ethics Committee of Sun Yat‐sen University.

## Consent

All authors agree to publish this article publicly.

## Conflicts of Interest

The authors declare no conflicts of interest.

## Data Availability

The data that support the findings of this study are available from the corresponding author upon reasonable request.
